# Identifying and fixing in-plane positioning and stability issues on a microscope using machine-readable patterned position scales

**DOI:** 10.1038/s41598-023-46950-y

**Published:** 2023-11-09

**Authors:** Olivier Acher, Matheus Belisario de Abreu, Alexander Grigoriev, Philippe de Bettignies, Maxime Vilotta, Thanh-Liêm Nguyên

**Affiliations:** 1 HORIBA France SAS, 14 Bd Thomas Gobert, 91120 Palaiseau, France; 2 HORIBA France SAS, 455 Avenue Eugène Avinée, 59120 Palaiseau, France

**Keywords:** Microscopy, Imaging, Optical imaging

## Abstract

Investigations of the in-plane positioning capabilities of microscopes using machine-readable encoded patterned scales are presented. The scales have patterns that contain absolute position information, and adequate software accurately determines the in-plane position from the scale images captured by the microscope camera. This makes in-plane positioning experiments simple and fast. The scales and software used in this study are commercially available. We investigated different microscopy systems and found that positioning performance is a system issue that is not determined solely by stage performance. In some cases, our experiments revealed software or hardware glitches that limited the positioning performance, which we easily fixed. We have also shown that it is possible to investigate vibrations using this approach and quantify their impact on image blurring. This is, for example, useful for experimentally determining the settling time after a stage movement.

## Introduction

The stability and accuracy of the in-plane position in microscopy are issues of increasing importance^1–4^. Observations can last for hours and extend overnight or over several days with modern fully automated microscopes driven by powerful software^5–7^. In this case, drift or inaccurate positioning may result in missing the Region of Interest (ROI) designated by the user or in image blurring. Modern microscope objectives make it possible to achieve a resolution of 200 nm according to Abbe’s law, and any drift or mispositioning comparable to or larger than this value will result in blurring or glitches. Single-molecule localization and super-resolution techniques^8, 9^ have pushed the resolution down by one order of magnitude and increased the need for stability in the 10 nm range, even though some position correction algorithms may mitigate stability issues^10, 11^. Microscopes may also provide an increasing number of modalities that are used sequentially, and positioning reproducibility is key to obtain accurate high-resolution multimodal images^12–14^. Correlative observations may also be performed on different instruments, and several methods have been developed to perform coordinate transfer between the coordinate systems of the two platforms^15–18^. Good positioning accuracy on both instruments is required to obtain good accuracy on the correlative observations.

In view of these increasing requirements for the stability, reproducibility, and accuracy of optical microscopes, several methods have been developed to perform experimental investigations on these subjects. Stability is commonly investigated by placing fiducials, such as fluorescent beads, in the field of a microscope and determining drift^10, 19^. This approach can also be used to determine the repositioning reproducibility at one position. Interferometers can be used to investigate stage performance with excellent precision, but their implementation on a microscope can be challenging. An additional limitation is that they provide the position in a referential external to the microscope, while the camera is the relevant referential. Another family of position-sensing techniques is based on computer microvision^20^ of machine-readable patterned position scales^21–30^. In this approach, a micropatterned scale contains encoded position information, and its images are decoded by appropriate software into in-plane positions and orientations. Recent developments have shown that out-of-plane coordinates can also be retrieved^25^. This general method has been implemented with different types of patterns, different algorithms, and imaging systems with widely different magnifications. It appears that this general method provides a resolution that overcomes the limitation due to pixel size by at least 2 orders of magnitude. Resolutions of less than 2 nm have been reported, which shows that the technique works in the super resolution regime. It has been used to perform in-plane position determination in various contexts, including robotics^23–25^, machine vision^30^, and microscopy^16, 21, 22^. Our group has developed a particular family of machine-readable patterned position scales and associated vision software, termed nanoGPS. We have already reported how to build 3 degrees of freedom absolute encoders using this approach^26^. We previously described how small multiscale multimodal nanoGPS-navYX tags can be used for coordinate transfer in correlative microscopy^16, 31^. In this paper, we present in detail the capability of machine-readable patterned position scale technology to determine the stability, reproducibility, and accuracy of a microscopy platform.

## Methods

**Figure 1:**
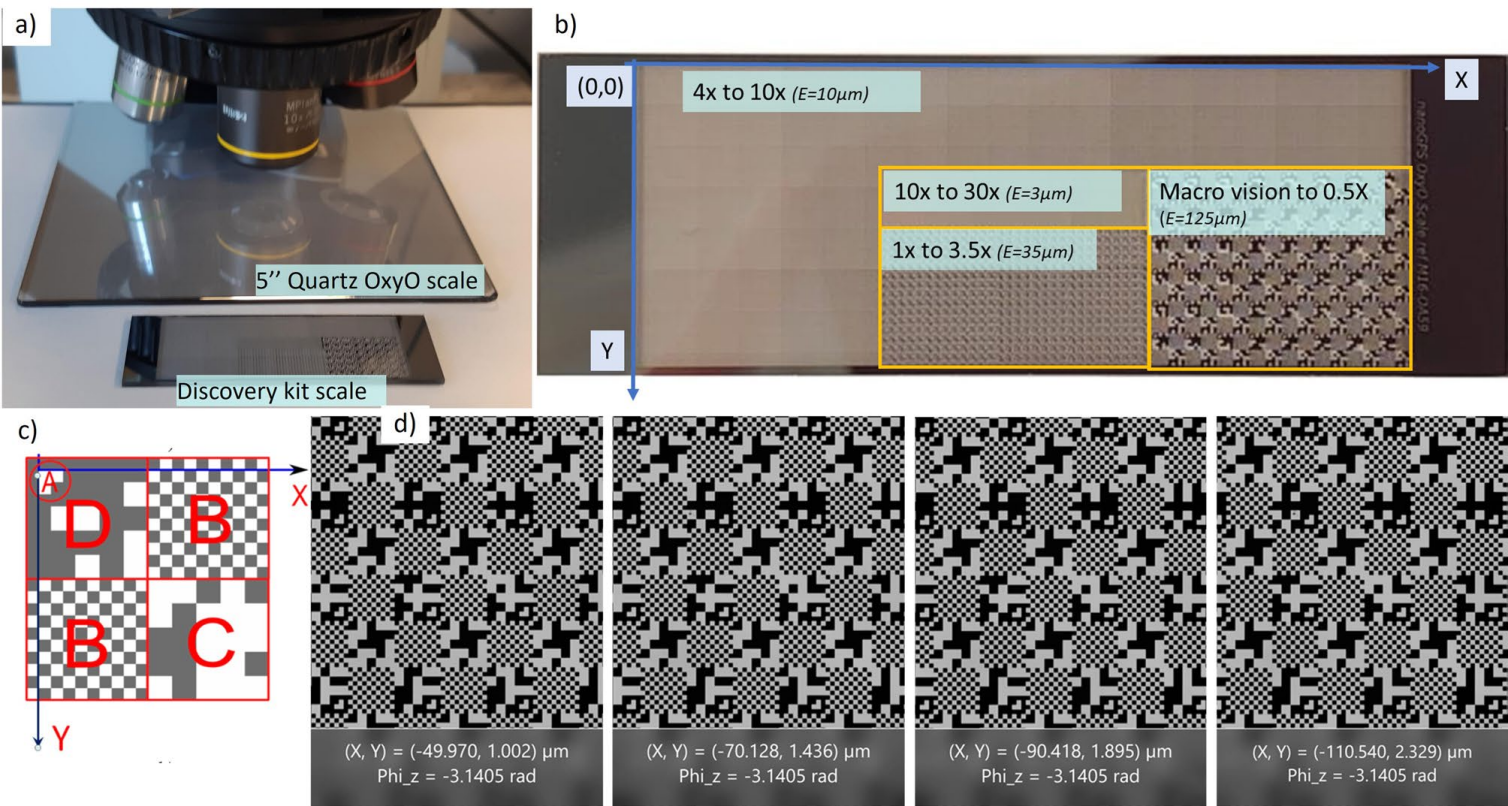
(**a**) nanoGPS OxyO scales used in the experiment; (**b**) layout of the discovery kit scale; (**c**) principle of absolute position coding in the scale; (**d**) sequence of images with corresponding nanoGPS coordinate extraction, with 20 µm translation stage movement between each image.

Two commercially available nanoGPS OxyO kits from HORIBA were used (Fig. 1a): one evaluation kit containing a scale with the same format as a microscopy slide^32^; and one 5-inch kit for 10 × vision system, Ultra High Accuracy^33^. The OxyO software that determines the positions and orientations from the nanoGPS images is included in each kit. The evaluation kit can be used with a wide variety of magnifications because it contains nanoGPS patterns at different scales (Fig. 1b). It can also be used with home-built micro or macrovision systems. The patterns are pseudoperiodic, and each pseudoperiod (Fig. 1c) contains an orientation pattern (A), chess patterns (B), and patterns in which position and scale references are encoded (C,D). The patterns are metallic and can be observed using a microscope either in reflection or transmission. The scale images have excellent contrast (Fig. 1d). The OxyO software can be applied to a folder of images to determine the corresponding trajectories.

We have previously reported a precision of approximately 1 nm and 10 µrad of the nanoGPS technology when used with a 10 × objective^26^, both for glass and quartz scales. In this study, we carried out stability and bidirectional reproducibility experiments indifferently with both scales, and 1 nm precision is more than enough for both conventional and super-resolution microscopes.

The positioning accuracy in X and Y depends on both the registration accuracy of the patterns on the scale and the thermal expansion of the scale between ambient and manufacturing temperatures. Registration accuracy is related to the fabrication technology, and thermal expansion is related to the scale material. In the case where the ambient temperature is in the [19.6 °C, 23.6 °C] range, the accuracy of the glass discovery kit slide for a 25 mm travel is estimated to be within ± 0.7 µm. The quartz scale has a much higher accuracy. It is an original photomask fabricated in a high-end facility that guarantees a registration accuracy better than ± 0.1 µm over 5 inches. Because of the low expansion coefficient of quartz, temperature variations in the range mentioned above would add another 0.1 µm uncertainty over 100 mm. Consequently, accuracy under laboratory conditions is expected to be better than ± 0.2 µm for a 100 mm travel. This has been confirmed experimentally through a collaboration with the metrology group of the Newport Micro-Controle company, and the results were presented at a conference dedicated to precision engineering^34^. A quartz 5-inch scale was used in all experiments related to stage positioning accuracy over multiple positions.

The in-plane orientation angle measured by the nanoGPS system has a precision better than 10 µrad, and an accuracy better than 63 µrad over 2π^27^.

The investigations were performed on several Nikon or Olympus upright microscopes equipped with different models of translation stages and placed on vibration isolation tables. They were equipped with 2 Mpixel global shutter monochrome cameras or 5Mpixel color rolling shutter cameras, all with a USB3 interface. Exposure time was chosen to avoid saturation. The image size was reduced to the field represented in Fig. 1d, which is sufficient to obtain excellent position determination while allowing a high frame rate and limited computational time to extract position information. A 10 × objective was used. Observations were carried indifferently with reflective (brightfield) or transmission illumination. High frame rate experiments (> 200 Hz) were carried out with transmission illumination because more light was available and the exposure time could be reduced to 3 ms.

This paper describes methods for investigating the in-plane positioning capability of microscopes, not to document this capability for particular setups. Therefore, we do not provide a full description of each microscope, stage, and vibration isolation table, along with their thermal and vibration environments.

A Python computer program was created to control stage movements and acquire images. Images were stored as bitmap files (.bmp) in one folder per experiment. After the experiment, the image folder was treated using OxyO software to create a file containing position information as a function of time. Stability and drift experiments were conducted by taking images at constant time intervals without introducing any voluntary stage movement. The vibration measurements were performed using an image maximum acquisition rate that ranged from 200 to 300 fps, depending on the illumination conditions and image size. The smallest image size required to obtain the nanoGPS position was determined using OxyO software.

**Figure 2:**
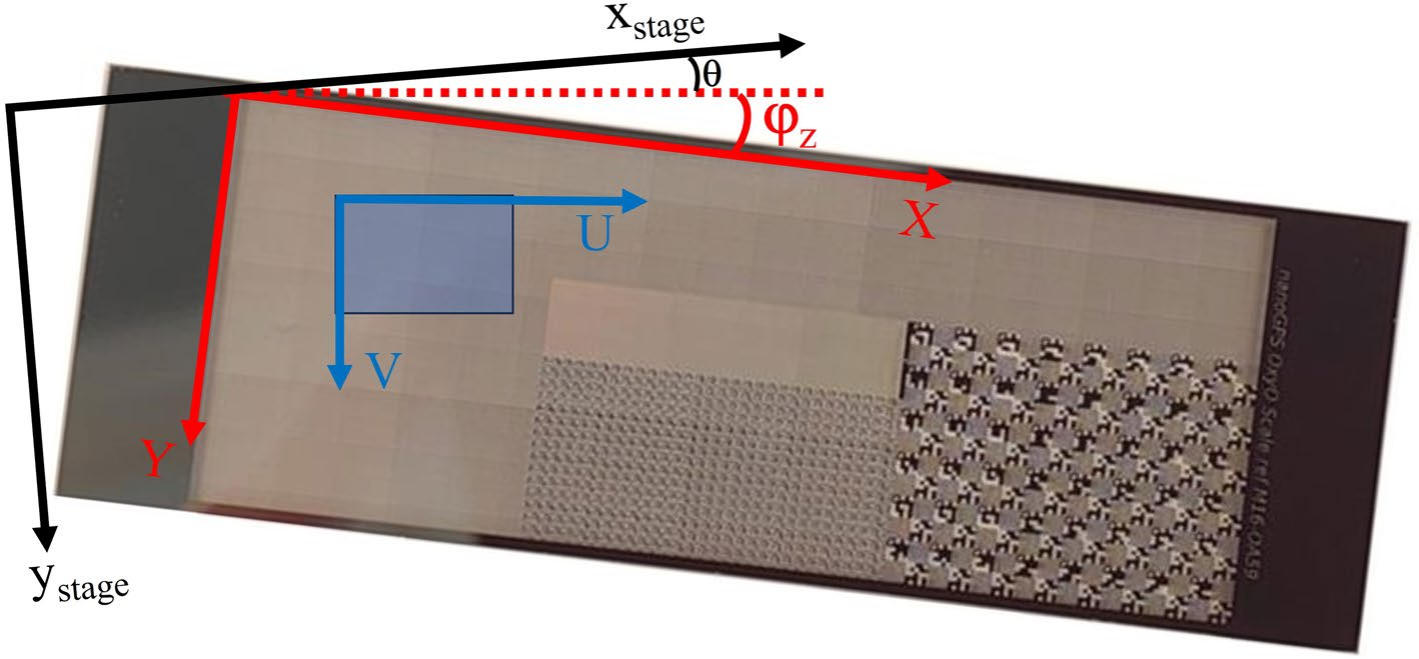
NanoGPS coordinates, camera coordinates and stage coordinates.

The trajectory provided by the OxyO software is expressed in the (O, X, Y) coordinate system of the scale. It is often more convenient to express it in the coordinate system of the camera (U,V axes), as shown in Fig. 2. The transformation of scale coordinates into camera coordinates is performed by applying the rotation by an angle φ_z_. As this angle between the scale and camera is determined by OxyO software anyway, this task could be solved very easily. We applied a rotation to (X, Y) coordinates by the constant average angle <φ_z_> to obtain the (U,V) coordinates.

It can also be very relevant to provide experimental results in the stage coordinate system when investigating stage properties. Even though the stage and camera often have nearly aligned axes, accurate measurements require the determination of the residual angle θ between the stage and camera. The angle [φ_z_ + θ] can be easily determined by instructing the stage to perform a large displacement along the x stage direction and determining ΔY/ΔX = tan(φ_z_ + θ).

For several experiments, validity assessments were performed by repeating the experiment after rotating the scale by 90°/180°/270°, and/or moving the scale by hand by a certain distance. In all cases, the measured positioning errors expressed in the stage referential were essentially the same. This simple check shows that the measured positioning errors are not related to any scale imperfections or any other limitation of the nanoGPS method. Even though the validity of the nanoGPS technique has been well documented, checking that it is working well with a new camera and a new optical setup can be a good practice.

## Results

The experimental results are organized as follows. First, we present investigations on stability performance. Then, we assess the performance of some microscopy stages. Bidirectional reproducibility related to a single position, accuracy, and reproducibility over more elaborate trajectories are reported. Next, we investigate vibration issues. We show that vibrations are not only related to the external environment but can also be intrinsic to the operation of the microscope. We present additional results in the Supplementary Data showing how to quantify the turret reproducibility, how to determine the scale bar in a very simple way, and how angle measurements can be useful.

### Stability

**Figure 3:**
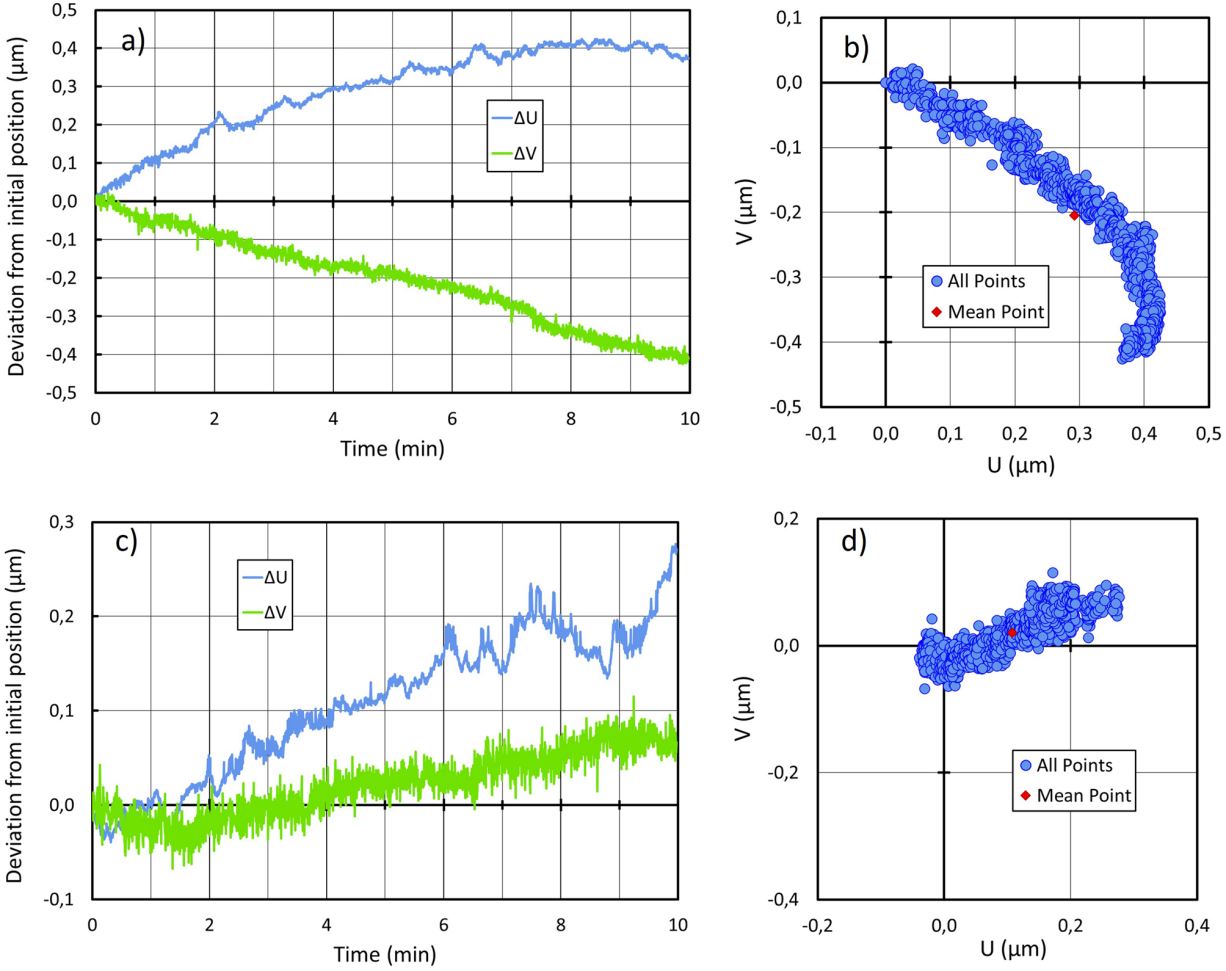
Coordinates of the central observation point as a function of time (**a**,**c**) and trajectory (**b**,**d**) over 10 min, recorded on a microscope at rest.

**Figure 4:**
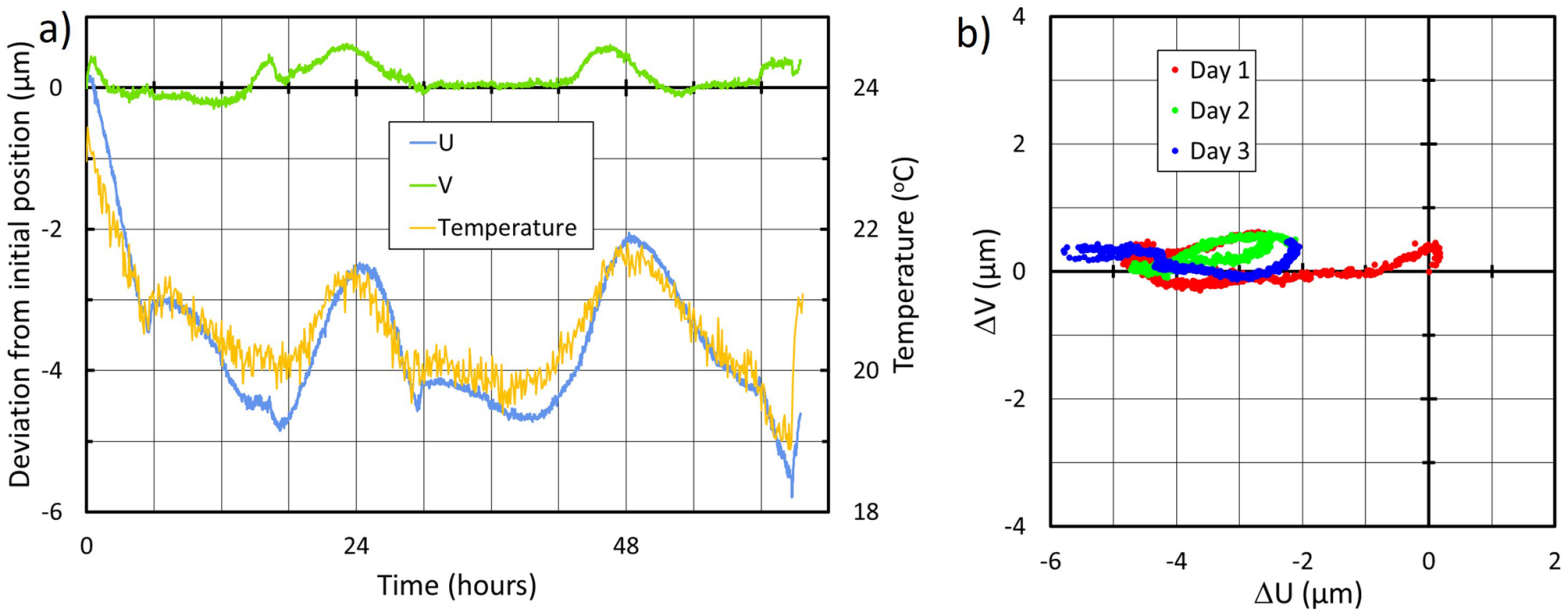
Records on a microscope at rest, over a weekend; (**a**) coordinates of the central observation point as a function of time, and lab temperature; (**b**) trajectory.

The stability was investigated over several timescales by recording the position of the nanoGPS scale in the camera referential: over 10 min at a frame rate of 30 Hz (Fig. 3), and over 68 h from Friday afternoon to Monday morning, at a frame rate of 2 images/min (Fig. 4).

Figure 3 shows that typical drifts over 10 min are commonly in the range of 0.1 to 0.4 µm. No obvious reason was found to explain that the two successive drift records differ significantly. The observation over 68 h (Fig. 4) reveals that the drift along the vertical direction of the image is less than 0.5 µm, but the drift along the horizontal direction can exceed 5 µm and is correlated to the lab temperature.

### Bidirectional reproducibility of a single position

**Figure 5:**
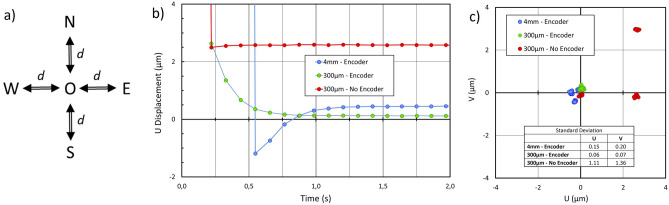
Determination of bidirectional reproducibility; (**a**) measurement principle: stage is instructed to return to point O, and the actual position is determined using nanoGPS, after visiting each N, S, W, E positions; cycle is repeated 20 times; (**b**) horizontal coordinate as a function of time, starting from position W at t = 0; (**c**) actual position after settling time, for d = 300 µm and d = 4 mm.

The determination of bidirectional reproducibility was inspired by a QUAREP-LiMi protocol^35^. Instead of using fluorescent beads, we used a nanoGPS scale. The stage was programmed to perform movements between point O and four cardinal points, situated at a distance d from O, repeated 20 times (Fig. 5a). Images of the scale were acquired at a 10 Hz frame rate. The settling time can be easily determined (Fig. 5b). The settling position is represented Fig. 5c. The stage has a built-in encoder that can be switched on and off. When it is switched off, the bidirectional reproducibility error can be as large as 3 µm in both directions. However, for a given direction of approach, the dispersion is very small, which suggests that if proper backlash correction is performed, bidirectional reproducibility can be excellent. When the encoder is turned on, the reproducibility is fine for d = 300 µm, but the settling time increases. The reproducibility slightly degrades for d = 4 mm.

### Accuracy and reproducibility of trajectories with different length scales

**Figure 6:**
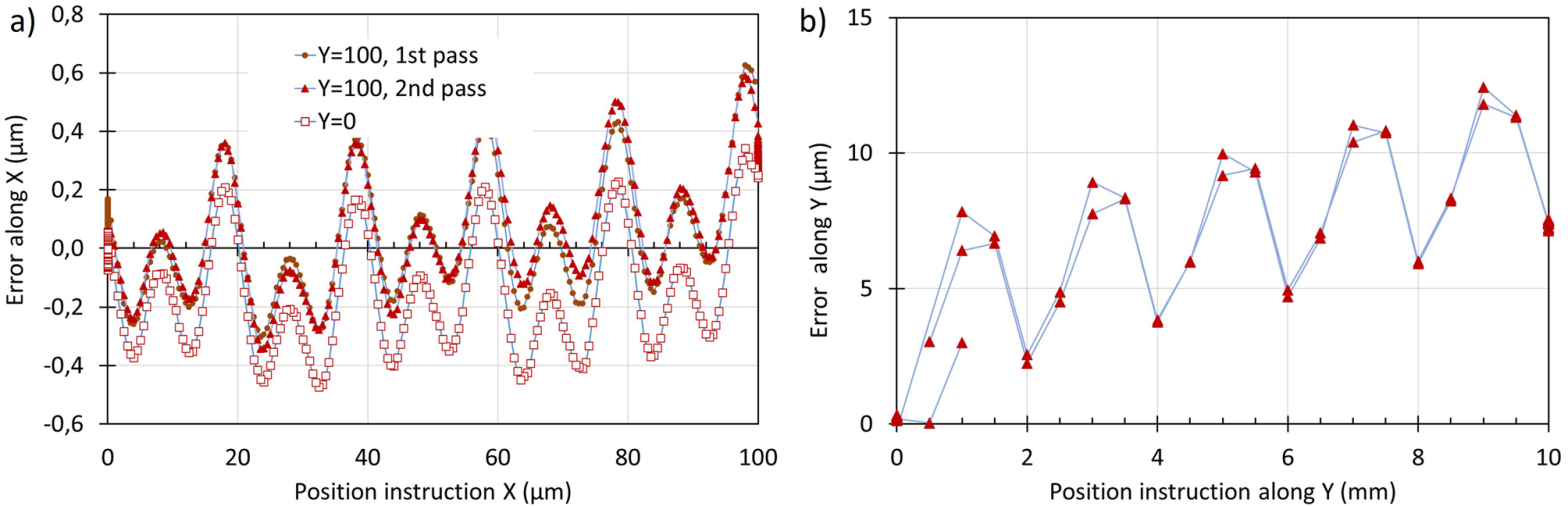
Positioning accuracy and repeatability observed for square trajectories repeated two times on a stage without encoder; (**a**) 100 µm square ; (**b**) 10 mm square.

The positioning errors observed on a stage without an encoder are shown in Fig. 6. Trajectories consisted of two successive squares, one with 100 µm side (Fig. 6a), and the other with 10 mm side (Fig. 6b). They reveal a repeatable error with 20 µm periodicity and 0.6 µm peak-peak value, and a repeatable error with 2 mm pseudo-periodicity and 5 µm amplitude.

**Figure 7:**
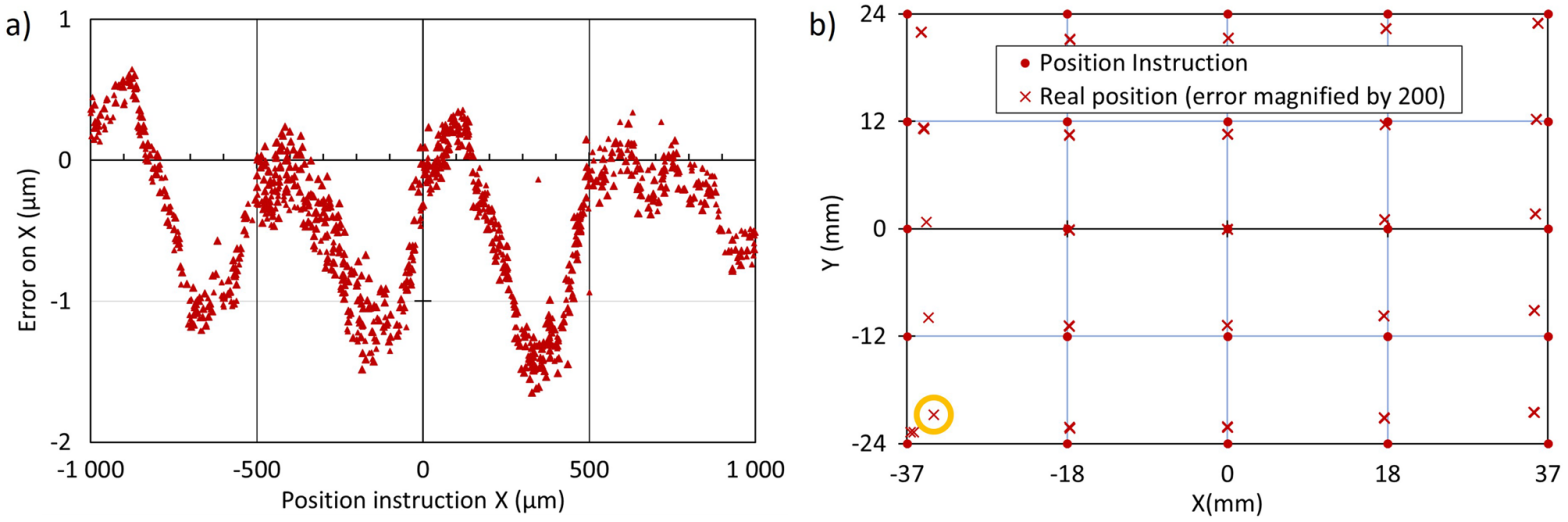
Positioning accuracy and repeatability observed on a stage with a magnetic encoder; (**a**) for random positions located within a 2 mm square; (**b**) on a 25 point grid, each point visited 3 times (maximum error is 15.3 µm along X and 17.7 µm along Y).

Figure 7 provides repeatability and accuracy data related to a stage with a magnetic encoder. In Fig. 7a, the data reveal a repeatable error with 0.5 µm periodicity and 1 µm amplitude, superimposed with an error that is not a repeatable function of X and has an amplitude of about 0.5 µm. The repeatability and accuracy over the full range of the table are shown in Fig. 7b. Reproducibility is rather good, except for one point in the corner (circled in orange). Accuracy is no better than 15 µm in X and 17 µm in Y.

The two figures above report positioning errors over trajectories with lengths encompassing 4 orders of magnitude, namely 0.1 mm; 1 mm; 10 mm; and 70 mm. We retained for display only two of the four families of trajectories for each type of stage. The ratio of the maximum error over the travel distance peaks at 10% for a 5 µm travel on Fig. 6a, 0.5% in Figs. 6b and 7a, and at 0.1% in Fig. 7b. We chose the ones with the most noticeable features because our purpose was not to perform a detailed evaluation of a particular stage model, but to explore the investigation method. Our results show that it is relevant to explore positioning error for trajectories over various travel lengths.

### Case of a heavy stage

**Figure 8:**
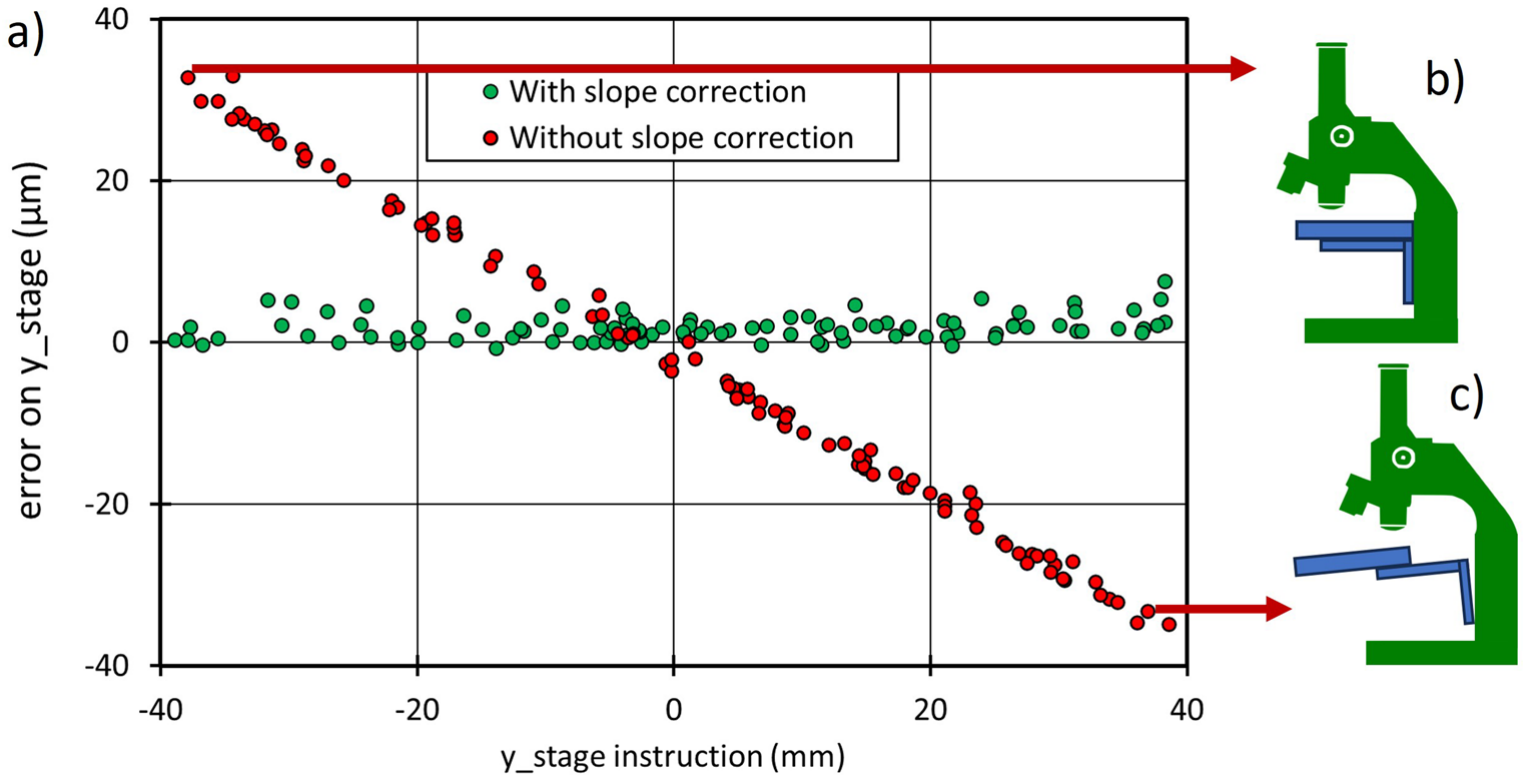
Case of a heavy large-area stage: (**a**) error on y_stage for random (x,y positioning) using manufacturer calibration (red), and after correction (green); (**b**) and (**c**): bending of the stage holder as the proposed cause for y_stage positioning error in the absence of correction.

The positioning accuracy of a microscope equipped with a large travel stage is shown in red in Fig. 8a. The positioning error along the y stage axis is linear in y and exceeds the manufacturer’s specifications. This error can be attributed to some bending of the mechanical parts that connect the stage to the main body of the microscope (Fig. 8b–c). This stage is much heavier than the stages with smaller travel. More importantly, the initial measurements can be used to tabulate a correction that is uploaded to the stage controller, and the position accuracy is improved (Fig. 8b, in green).

The presence of an autofocus mechanism was essential for acquiring images of the nanoGPS scale because of significant defocus when the stage was moved in the y direction. This defocus effect is one of the damaging effects of insufficient rigidity of the microscope body and stage holder, and it is not corrected by the approach presented here.

### Vibrations

**Figure 9:**
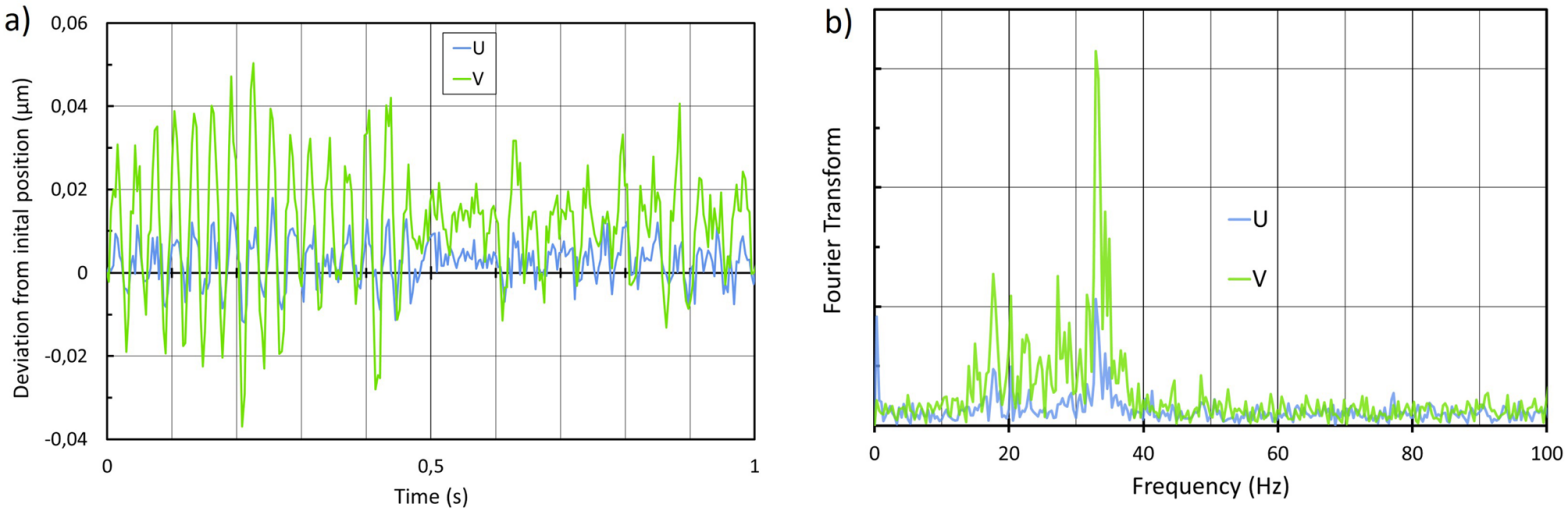
Position stability of the microscope image over a few seconds: (**a**) coordinates of the central observation point in the (U,V) referential of the camera as a function of time; (**b**) Fourier transform of (**a**).

**Figure 10:**
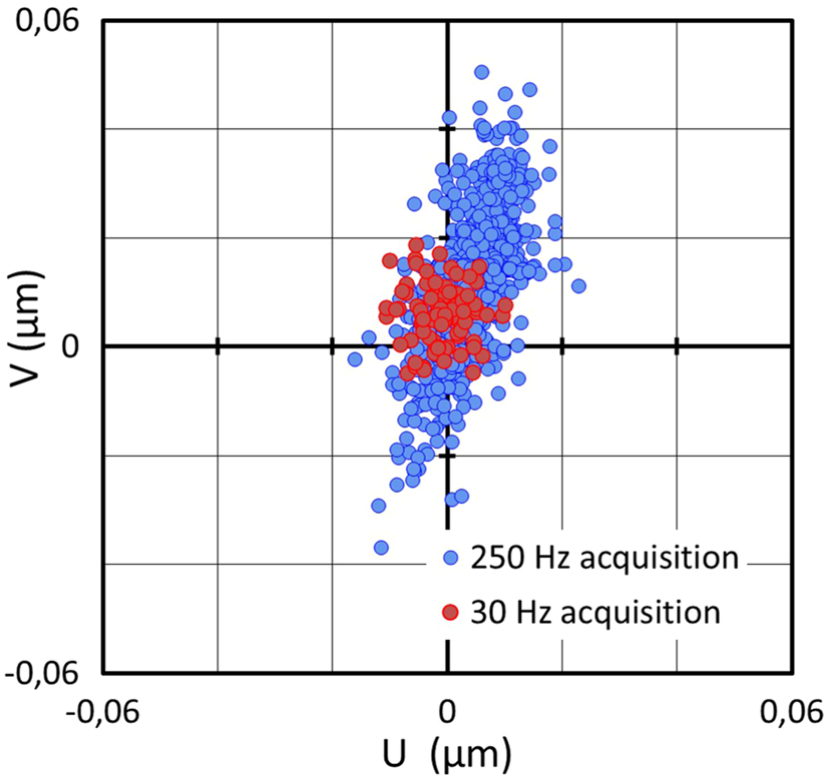
Position stability of the microscope image over a few seconds, measured with 3 ms exposure time and high frame rate (blue), and with 30 ms exposure time and lower frame rate (red).

The acquisition of nanoGPS scale images at frame rates larger than 200 Hz can be performed using transmission illumination. The results of a stability experiment are presented in Fig. 9, and reveal a 80 nm peak-peak instability along one direction. The Fourier analysis in Fig. 9b shows that strong resonance at a frequency close to 35 Hz is responsible for this behavior. Figure 10 shows both the trajectory corresponding to the high-frequency measurement (in blue) and the trajectory recorded at 30 Hz, with a 30 ms exposure time. The noise on the position measured at 30 Hz appears to be significantly lower than that at 250 Hz.

**Figure 11:**
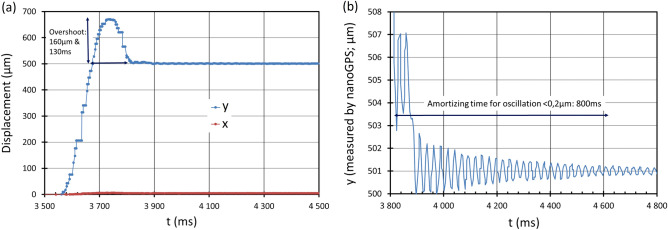
NanoGPS recording of stage position after instruction to perform à 500 µm movement along Y: (**a**) observed from start (**b**) observed during the stabilization period.

The transient position of a stage equipped with an optical encoder was also investigated. Figure 11 shows the position of the stage determined by nanoGPS after it received the instruction to move by 500 µm along Y. Some images were too blurred to be decoded and returned an error: the corresponding points are represented on the graph as “the same position as former point”. However, most of the stage trajectory can be monitored very adequately. Two main observations can be made. As evidenced in Fig. 11a, there is an overshoot of the stage movement along Y. Figure 11b provides a closer view of the stage movement after it nearly reached its final position. A vibration on the position is clearly evidenced, with a frequency of 39 Hz and an initial peak-to-peak amplitude exceeding 2 µm. The vibration is damped, but it takes 0.8 s for its amplitude to go below 0.2 µm. The stage encoders do not reveal such vibrations, indicating that the observed vibration originates from the structure of the microscope and not from the stage.

Supplementary Data illustrate further situations in which the experimental determination of vibration using nanoGPS OxyO provides useful information. This study also illustrates further uses of this technology, including turret reproducibility experiments, determination of the scale bar through a single OxyO measurement, determination of the residual angle between the stage and camera, and determination of the yaw angle of the stage.

## Discussion

Stability experiments reported in Figs. 3 and 4, along with the vibration measurements reported in Figs. 9 and 11 and in Supplementary Data, show that the technique presented here can be effective in assessing whether the microscope system and its environment may be a limitation for a given type of experiment. The answer will depend on the measurement types the experimentalist wants to carry and will differ if the microscope is used in a diffraction-limited configuration or in a super-resolution configuration, if visiting the same points of interest is part of the process, etc.… Inadequate system or environment may result in image deformation, blurring, registration errors and inconsistencies. Interestingly, these experiments can also provide simple clues to avoid such effects without resorting to over-specified and costly solutions. Measurement such that of Fig. 4 and Supplementary Fig. 1 provide a sound basis for deciding whether to invest in tighter climate control or antivibration tables.

We have also shown that the positioning capability of the microscopy stages can be evaluated using this technique, as presented in Figs. 6, 7 and 8. The large quartz scale used in our experiment has an accuracy that meets or may exceed the requirements for microscopy accuracy. The periodic error in Fig. 6a is attributed to inaccuracies in the microsteps of the electrical motor that drives the X spindle and that in Fig. 6b to the periodicity of the worm screw. The periodic error in Fig. 7a is due to the imperfection of the magnetic encoder. Errors greater than 10 µm on Fig. 7b may seem large for a stage with an encoder. However, neither the calibration temperature of the stage nor the laboratory temperature during the experiment are known, and 10 µm is the thermal expansion of a 150 mm long piece of aluminum for a 3 °C temperature increase. Our experimental results also suggest that the X-rail guide may have some curvature.

Our results also indicate that positioning accuracy should be determined under actual experimental conditions. The specification sheet and quality control results issued by stage manufacturers are useful. However, our results in Figs. 3 and 4 show that the microscope body drift may also induce significant positioning errors that should not be attributed to the stages. Figure 8 shows that the way the stage is mounted may also introduce errors. Supplementary Fig. 3 illustrates that activating the turret may also affect objective decentering. Software settings also contain many pitfalls. During our experiments reported in Fig. 5, we first obtained the results labelled “no encoder”, while we expected that our stage would perform much better because it was equipped with an encoder. After suspecting some encoder malfunction, we found that the encoder was not activated due to a software issue with the stage controller, and we corrected it. We also found that we could remove the overshoot shown in Fig. 11a by adequately setting some software parameters. We also found hardware glitches on different microscopes, such as numerous issues with stage or camera cables inducing position shifts when inadvertently moved or stressed; and poor accuracy on a microscope with a stage fixation screw loosened. It may also be useful to experimentally determine the settling time because, as illustrated in Fig. 5 and Fig. 11, the fact that the stage believes it has reached its final position does not mean that the image is stabilized on the camera, and images taken without respecting the settling time may be blurred.

Blurring can be a consequence of vibrations^36^, and we have shown in Figs. 9, 10, 11 and Supplementary Data that vibrations are a system-level issue. The experimental approach presented here makes it simple to detect vibrations up to 100 Hz, and to test solutions that limit the vibrations to an acceptable peak-peak amplitude in the image plane.

One limitation of the approach presented here is that position sensing is not performed simultaneously with sample observations. Therefore, it cannot be used for real-time correction. The reason is that the same camera is used for both scale and sample visualization. Performing sequential observation of the sample and of the scale by moving the z-focus from the sample to a patterned coverslip has been demonstrated^21, 22^, and may be very adequate to render long experiments immune to drift. Alternatively, an additional camera can be used to read the scale. If the system is designed such that deformations of the microscope body similarly affect the main and additional cameras, this could lead to superior position encoders integrated in the microscope body.

## Conclusion

We have shown that machine-readable encoded patterned scales are convenient for investigating positioning accuracy and stability issues on both conventional and super-resolution microscopes. It is based on imaging a patterned scale with the microscope camera. Like previously developed techniques based on imaging fiducials and fluorescent beads developed to determine stability and reproducibility, it provides the position in the referential of interest, which is the camera. Because the entire surface of the slide can be used, it also allows absolute position measurements at any position without special preparation. We showed that position measurements could be performed up to frame rates exceeding 200 Hz, which is very useful for investigating vibrations that may cause either image instability or blurring.

Another conclusion of our work is that positioning stability, reproducibility, and accuracy should be performed on microscopes in their real operating conditions and environments because it is not only an issue related solely to the moving stage but also a system issue. Whether the position capability of a microscope is sufficient for a given experiment can be easily determined by programming the microscope and stage movement according to the experiment and placing a machine-readable encoded patterned scale instead of the sample. The scale images obtained during the dummy experiment provide the actual trajectory. The comparison of the measured trajectory with the requirements of the experiments and the vibration amplitude with the required resolution can provide a direct indication to the experimentalist of any impacting limitation of the positioning capability. This experimental approach not only makes it easy to detect and solve software or hardware glitches that may undermine the microscope positioning performance, but also quantitatively defines the requirements for more expensive modifications such as temperature or vibration control.

### Supplementary Information

Supplementary Information.

## Data Availability

The datasets analyzed in this study are available from the corresponding author upon reasonable request.
