# Supplementary material for: Identifying and fixing in-plane positioning and stability issues on a microscope using machine-readable patterned position scales
Source: Sci Rep. 2023 Nov 9;13:19521. doi: 10.1038/s41598-023-46950-y (PMC10636118; doi:10.1038/s41598-023-46950-y)
Supplement: Supplementary file 1 — Supplementary Information. [file 41598_2023_46950_MOESM1_ESM.docx]

# Supplementary Data

## *S.1 Vibrations*

High-frequency position measurements using nanoGPS OxyO® can be used to determine whether the vibration levels are sufficiently low. Supplementary Fig. 1 shows the influence of footsteps on the laboratory close to the microscope. The microscope was placed on a vibration-insulation table. We first observed (Supplementary Fig. 1a) that footsteps created a vibration with 1µm peak-peak amplitude, which can affect the image quality. We found that one of the four air suspensions of the table had insufficient air pressure, and after correcting this issue, we found that footsteps do not impact image quality, as shown in Supplementary Fig. 1b.


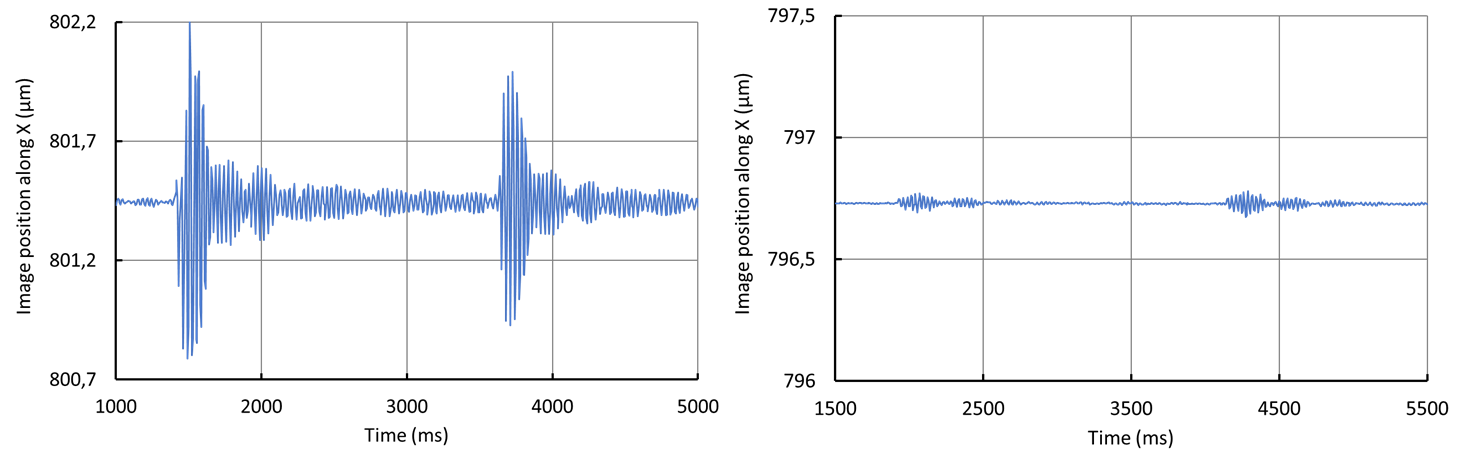


(a) (b)

*Supplementary Fig. 1. nanoGPS recording of X position during two footsteps of an operator close to the microscope: (a) with badly installed vibration insulation table (b) with efficient vibration-insulation table.*

Even in the case where the vibration table has excellent efficiency, actions internal to the microscope can create vibrations that may blur the microscope image. Supplementary Fig. 2 shows the effect of switching a shutter integrated in the microscope. The amplitude of the movement is at most 150nm peak-peak, which is negligible on a conventional microscope. On a super-resolution microscope, the operation of the shutter may affect the quality of the images by creating a blur exceeding 20nm for about 1,5s.


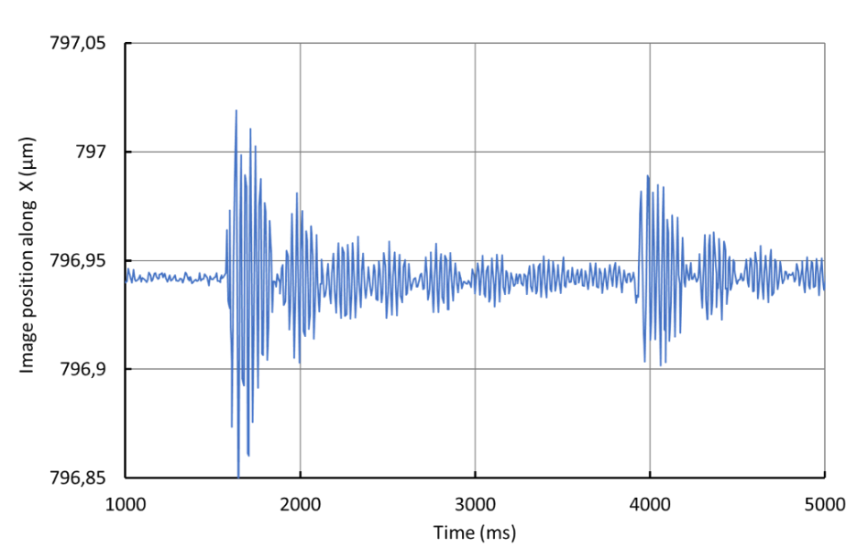


*Supplementary Fig. 2. nanoGPS recording of X position during two operations of a shutter integrated in the microscope.*

## *S.2 Turret reproducibility experiments*

The reproducibility of a turret can be quantified using nanoGPS OxyO®, as illustrated in Supplementary Fig. 3.


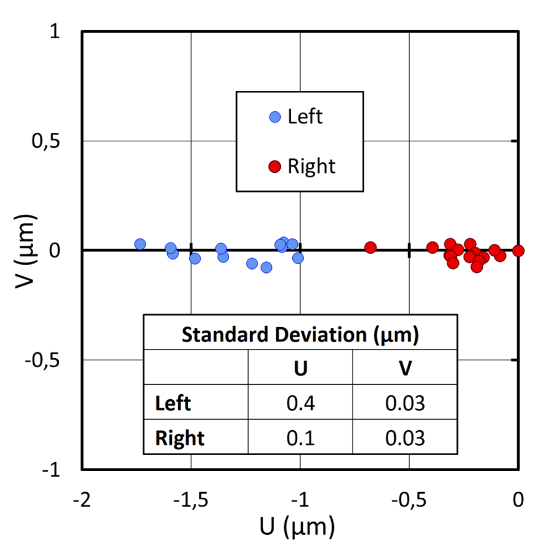


*Supplementary Fig. 3. Position of the image center after maneuvering the turret in two directions.*

## *S.3 Determination of the scale bar objective magnification*

The OxyO software accurately determines the period of the chessboard patterns shown in Fig. 1c, and this period is known very precisely because of the high manufacturing accuracy of the nanoGPS scales. The OxyO file stores the pixel size in the sample plane (expressed in µm/px), which make it very convenient to create accurate scale bars on images.

Supplementary table I reports the precise determination of the scale using the OxyO kit scale, which is suitable for determining the scale for different objectives, using various regions of the scale (Fig. 1b). A single image of the scale treated by OxyO software is sufficient to obtain scale bar information. To check the consistency of our results, we determined the pixel size in the sample plane from two different zones of the scale for most of the objectives and found that the results are perfectly consistent, as shown in Supplementary Table I.

*Supplementary table I. Determination of magnification using nanoGPS OxyO software for different objective lenses using different nanoGPS scale zones.*

## *S.4 Angle determination*

NanoGPS OxyO also returns angle information. Optical microscopes are generally equipped with a translation stage, and by definition, a translation, one would expect the angle between the scale and the camera to be independent of the position. Supplementary Fig. 4 shows that this is the case for the microscope under investigation, within +/-200µrad, except close to one extremity of the stage movement, where a yaw up to 2mrad is observed.

Perhaps a more important application of the angle measurement is to be able to determine (as mentioned in the “methods” section) the angle between the stage and camera, which can be useful for performing sample mapping over large areas.


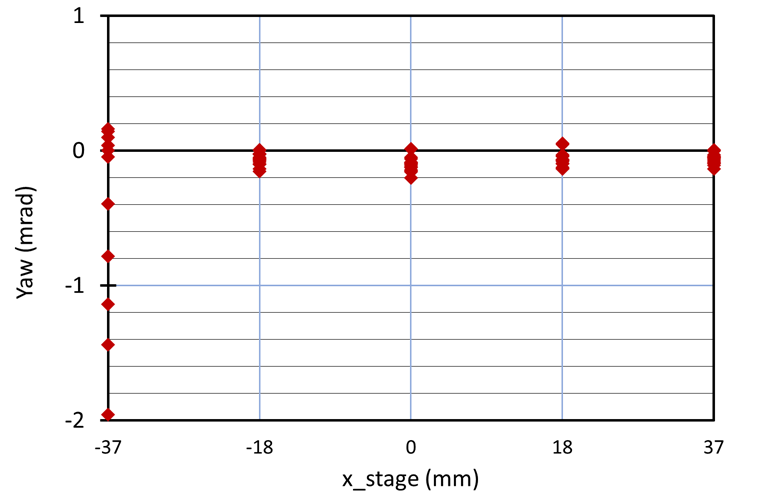


*Supplementary Fig.4. Yaw angle determined from nanoGPS experiments, for the experiments reported in Fig. 7b.*
